# The tumor suppressor archipelago E3 ligase is required for spermatid differentiation in Drosophila testis

**DOI:** 10.1038/s41598-021-87656-3

**Published:** 2021-04-19

**Authors:** Viktor Vedelek, Attila L. Kovács, Gábor Juhász, Elham Alzyoud, Rita Sinka

**Affiliations:** 1 Department of Genetics, University of Szeged, Szeged, Hungary; 2 Department of Anatomy, Cell and Developmental Biology, Eötvös Lóránd University of Science, Budapest, Hungary

**Keywords:** Development, Germline development

## Abstract

The human orthologue of the tumor suppressor protein FBW7 is encoded by the Drosophila *archipelago (ago)* gene. Ago is an F-box protein that gives substrate specificity to its SCF ubiquitin ligase complex. It has a central role in multiple biological processes in a tissue-specific manner such as cell proliferation, cellular differentiation, hypoxia-induced gene expression. Here we present a previously unknown tissue-specific role of Ago in spermatid differentiation. We identified a classical mutant of *ago* which is semi-lethal and male-sterile. During the characterization of *ago* function in testis, we found that *ago* plays role in spermatid development, following meiosis. We confirmed spermatogenesis defects by silencing *ago* by RNAi in testes. The *ago* mutants show multiple abnormalities in elongating and elongated spermatids, including aberration of the cyst morphology, malformed mitochondrial structures, and individualization defects. Additionally, we determined the subcellular localization of Ago protein with mCherry-Ago transgene in spermatids. Our findings highlight the potential roles of Ago in different cellular processes of spermatogenesis, like spermatid individualization, and regulation of mitochondrial morphology.

## Introduction

*Drosophila melanogaster* testis is a particularly suitable model to follow the process of spermatogenesis^1,2^. During Drosophila spermatogenesis from a single spermatogonia sixty-four sperms are being produced, through mitotic and meiotic cell divisions and intensive cellular remodeling. After each division, the daughter cells remain in connection with plasma bridges, which allows them to develop simultaneously in a syncytial cyst. The early transit-amplifying mitotic divisions result in sixteen spermatocytes. Spermatocytes undergo a maturing process, they have high transcriptional activity until meiosis and the accumulated transcripts contribute to the development of the transcriptionally mostly inactive post-meiotic stages^3^. After meiosis the mitochondria aggregate and fuse to establish the nebenkern structure, which consists of two mitochondrial derivatives. Next, the spermatids start to elongate to reach a full length of ~ 1.8 mm, which approximately 150 times longer than their initial diameter. The two mitochondrial derivatives run along the axoneme in the spermatid's tail and progressively differentiate. One of them becomes the major derivative with paracrystalline material accumulation, while the other one loses its volume and becomes the minor mitochondrial derivative. As part of the spermatid maturation, the nuclei also elongate while their volume decrease as the chromatin structure condensates and histones are replaced by protamines. Individualization, the process which establishes the individual sperms starts with the formation of the individualization complex (IC), which contains the F-actin-rich cone-shaped investment cones that are forming around the elongated nuclei. The IC migrates from the nuclei towards the tail end of the cyst, during its progression the majority of the cytosol and its content are displaced. An individual membrane starts to develop around each spermatid and a cystic bulge emerges. A non-apoptotic caspase activity facilitates the process of individualization^4^. The caspase activity is mainly restricted to the cystic bulges and later in the waste bags at the end of the individualization^4,5^.

Remodeling of the round spermatocytes to functioning sperms is requiring strict regulation, selective protein degradation, and sufficient proteasome activity^6^. There are multiple ubiquitin ligases known to have a role in the different steps of spermatogenesis (e.g.: Parkin, Ntc/Cul1/SkpA-SCF^Ntc^)^7,8^, and testis-specific proteasome subunits are emphasizing the ubiquitin–proteasome system’s (UPS) role^9^.

E1, E2, and E3 orchestrate the ubiquitination of proteins inducing their degradation. While there are only a few types of E1 and E2 enzymes, many E3 enzymes were identified^10,11^. E3 ligases can mark proteins for time and tissue-specific degradation therefore they have a role in many different cellular processes such as cell cycle, epigenetic regulation, mitophagy, etc.^12^. Two E3 ligase complexes are necessary to activate the non-apoptotic caspase cascade, the Klhl10/Cul3/Roc1b and the Ntc/Cul1/SkpA E3 complexes during individualization of spermatids^8,13^. The F-box protein Archipelago (Ago) is a member of an evolutionary conserved E3 ligase complex (SCF type), as the subunit responsible for substrate specificity of the complex^14–16^. The human orthologue of *Ago* is the FBW7 (FBXW7, hCDC4, hAgo, hSel-10), which is a known tumor suppressor^17–23^. *Drosophila* Ago is well characterized in the neuronal system, embryonic trachea, eyes, and during oogenesis, and it has interaction with dMyc, Cyclin-E, Notch and Trachealess^15,16,24–27^.

Here we present the role of the pleiotropic Ago protein, during the post-meiotic stages of *Drosophila* spermatogenesis. We show that lack of Ago resulted in male sterility and abnormal individualization of spermatids, including abnormal nuclear and mitochondrial structure.

## Results

### Lack of* ago* cause male sterility in Drosophila

**Figure 1:**
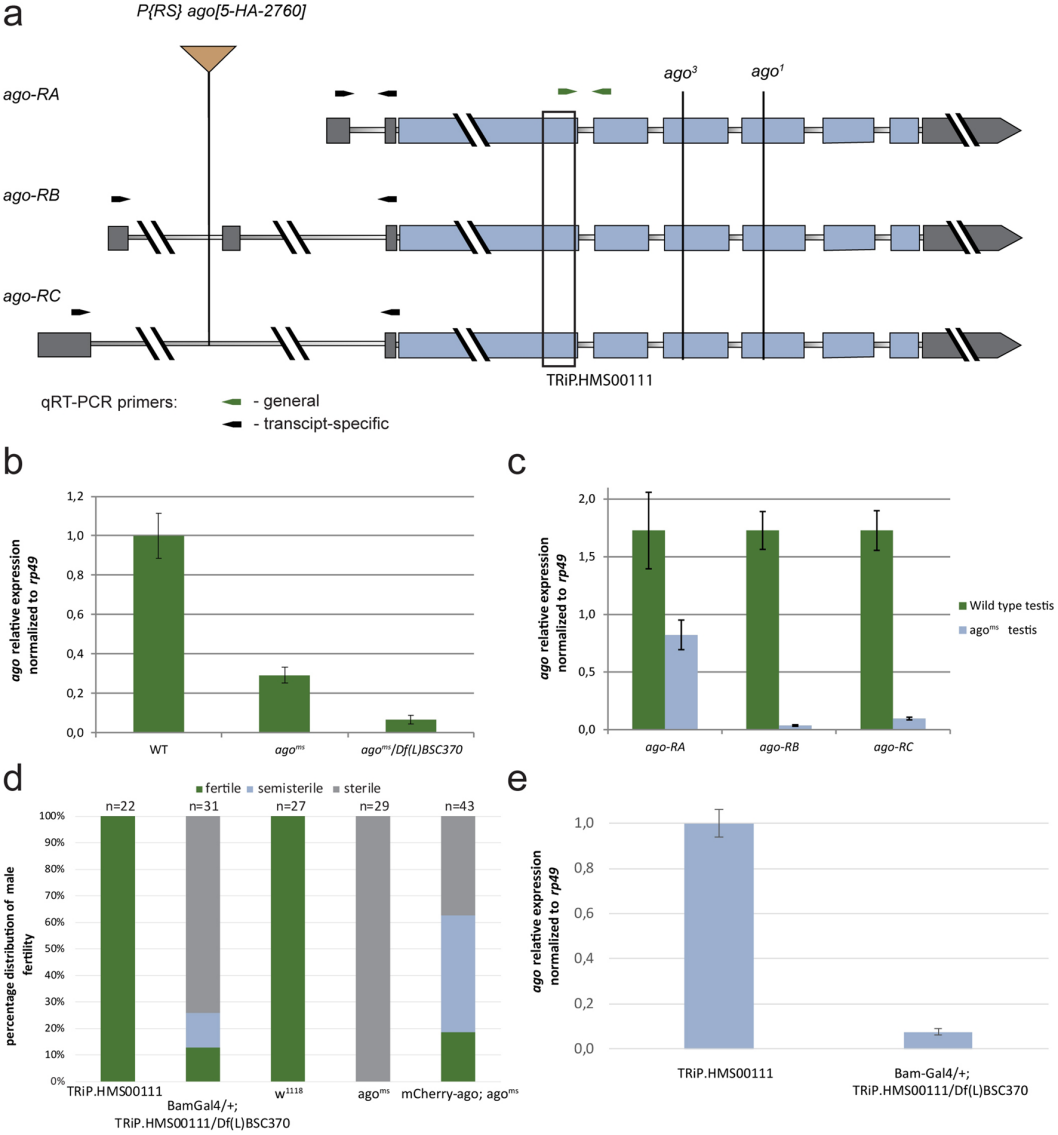
Fertility in *ago* mutants and *ago* expression in testis. (**a**) The schematic structure of *ago* transcripts. Thin bars indicate the intronic regions, thick grey bars indicate the UTR sequences and thick blue bars indicate the protein-coding sequence of *ago*. P{RS}-*ago*^*ms*^ element insertion is annotated at the 5′ UTR intronic region of *ago-RB* and *ago-RC* transcripts. EMS mutant *ago*^*1*^ and *ago*^*3*^ are labelled. The black bracket represents the gene region targeted by the dsRNA in the *ago*^*TRiP*^ line. Arrows indicate quantitative RT-PCR primers, the green arrows represent the *ago* coding sequence-specific primers, and the black arrows indicate the *ago* transcript-specific primers to the 5′UTRs. (**b**, **c**) Relative total and isoform-specific *ago* transcript levels were normalized to *rp49* in wild type (WT), *ago*^*ms*^*/ago*^*ms*^*,* and *ago*^*ms*^*/Df BSC370*. (**d**) Reduced fertility was observed in *Bam-Gal4/* + ; *TRiP.HMS00111/ Df(L)BSC370* males, compare to *TRiP.HMS00111* (control line without the driver) males. mCherry-Ago rescues partially the male-sterile phenotype of *ago*^*ms*^ (**e**) Relative expression levels of total *ago* transcripts in + / + ;*TRiP.HMS00111/* + control and *Bam-Gal4/* + ; *TRiP.HMS00111/Df(L)BSC370* testes were normalized to *rp49*. Vector graphics were created in Adobe Illustrator CS6 ver. 16.0.3. Charts were created in Microsoft Excel 2016 MSO ver. 16.0.4266.1001 and processed in Adobe Illustrator CS6 ver. 16.0.3.

We identified a male-sterile allele of the a*rchipelago* gene, *ago*^*5-HA-2760*^ (*ago*^*ms*^). In *ago*^*ms*^ there is a P{RS5}-element insertion in the intronic region of the 5′ UTR of the *ago* gene (Fig. 1a). We tested genetically a*go*^*ms*^ mutant line and found that *ago*^*ms*^ is semi-lethal and male-sterile in homozygous and in hemizygous combination with the overlapping *Df(3L)BSC370* deficiency and lethal in transheterozygous combination with the previously described *ago*^*1*^ and *ago*^*3*^ alleles^24^. These results suggest *ago*^*ms*^ is a strong hypomorph allele. Precise excision of the P-element in *ago*^*ms*^ restored wild type fertility. Next, we investigated how *ago* transcripts could be affected by the transposon insertion. We performed quantitative RT-PCR using mRNA samples of wild type and *ago*^*ms*^ testes to measure the levels of *ago* transcripts. Three different *ago* transcripts are annotated, however, a single polypeptide is encoded by *ago* (Fig. 1a). We found a strong decrease of *ago* mRNA level in *ago*^*ms*^ mutant homozygotes and hemizygotes measuring the three *ago* transcripts together (Fig. 1a,b). When we tested the *ago* transcripts individually and we found a dramatic reduction of *ago-RB* and *ago-RC* transcript levels and also a moderate reduction of *ago-RA* transcript level (Fig. 1a,c). High througput experiments reported the *ago* transcripts mainly enriched in the apical region but present in later stages as well^9,28^. To investigate the expression profile of the *ago* isoforms we isolated RNA from the apical and basal parts of the testis. We used *CG3927* as an apically and *CG10252* as a post-meiotically enriched controls, and normalized the gene expression to *rp49* (Fig. S1 a)^3,29^. The RT-qPCR results shows the presence of *ago* transcripts both in the apical and basal parts, and suggests a more stable transcript level than *CG3927* and *rp49*.

To study the role of Ago exclusively during spermatogenesis, without the potential somatic influence^30^ and to overcome the semi-lethality of the *ago*^*ms*^ mutant, we utilized the *P{TRiP.HMS00111}attP2ago* (*ago*^*TRiP*^) transgenic RNAi line. *ago*^*TRiP*^ was driven by the germline-specific *Bam-Gal4* driver in the presence of *Df(3L)BSC370* deficiency (*ago*^*TRiPDf*^) to increase the silencing effect (Fig. 1a). We used the driverless + */* + *; Df(3L)BSC370/ P{TRiP.HMS00111}attP2ago* flies as control unless we state otherwise. RNAi knockdown of *ago* recapitulates the classical mutant phenotype, resulting in ~ 70% of sterility in the male offspring (Fig. 1d). We confirmed the downregulation of *ago* transcripts with quantitative RT-PCR in *ago*^*TRiPDf*^ testis (Fig. 1e).

### Disrupted spermatid individualization in *ago* mutant testes

**Figure 2:**
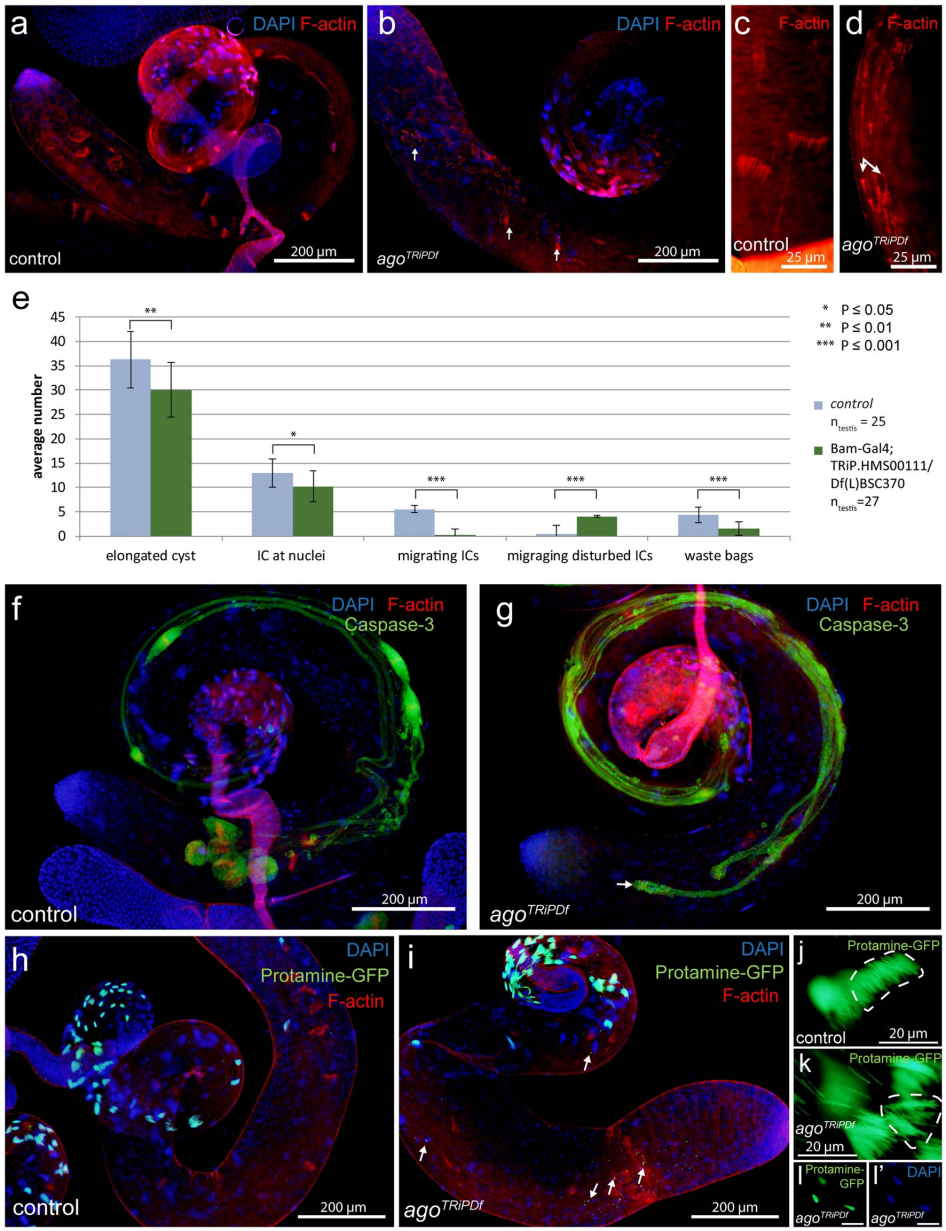
Individualization defects in *ago*^*TRiPDf*^ testes and Protamine-GFP (green) accumulation in *ago*^*TRiPDf*^ spermatids. (**a-d**) Investment cones visualized by fluorescent microscopy using Texas Red-X phalloidin staining (red) and nuclei with DAPI in control (**a**,**c**) and *ago*^*TRiPDf*^ testes (**b**,**d**). Scattered investment cones with variable size are present in the *ago*^*TRiPDf*^ cysts (**b**,**d** arrows). (**e**) The average number of individualization complexes (ICs) in control and *ago*^*TRiPDf*^ testis. Error bars indicate mean ± s.e.m. Statistical significance was determined by Welch two-sample t-test. (**f-g**) Anti-cleaved Caspase 3 (green) highlight the caspase activity in the control and *ago*^*TRiPDf*^ cysts. Morphologically normal cystic bulges and waste bags are present and cleaved Caspase3 accumulates in control testes (**f**). In *ago*^*TRiPDf*^ testes (**g**), the cystic bulges and waste bags are severely damaged. (**h–l’**) Nuclei are visualized with Protamine-GFP (green) and DAPI (blue), F-actin with Phalloidin-Texas Red (red). Nuclear bundles are slightly disoriented in *ago*^*TRiPDf*^ cyst compared to wild type (**h,j**, **i,k**). DAPI and Protamine-GFP positive hypercondensed nuclei are present in *ago*^*TRiPDf*^ testes (**i** arrows, **l,l’**). The chart was created in Microsoft Excel 2016 MSO ver. 16.0.4266.1001 and processed in Adobe Illustrator CS6 ver.

To understand the function of Ago during spermatogenesis we analyzed the morphology of both *ago*^*ms*^ and *ago*^*TRiPDf*^ testes (Supplementary Fig. S1 b-g’, Fig. 2a,b,f,g,h,i). The seminal vesicles are empty in the *ago*^*ms*^ mutants, but cyst elongation occurs after meiosis, which suggests that the elongated spermatids are failed to individualize (Supplementary Fig. S1 b,c). Visualizing DNA with DAPI and investment cones with Texas Red-X phalloidin staining revealed that individualization is disturbed in both *ago*^*ms*^ and *ago*^*TRiPDf*^ testes (Supplementary Fig. S1 d-g’, Fig. 2a–c,f–i). While investment cones are simultaneously migrated through the length of the elongated cyst in wild-type, their movement becomes misdirected, asynchronous and they are scattered by variable size along in both the classical and the *ago*^*TRiPDf*^ cysts. (Supplementary Fig. S1 f, g,g’, Fig. 2c,d,e).

We visualized the activation of the Caspase cascade with α-cleaved Caspase-3 antibody staining. In wild type testis, active-Caspase-3 signal mostly restricted to cystic bulge and the preindividualised parts of the spermatids and finally accumulate in the waste bags following individualization (Fig. 2f,g). The asynchronous migration of actin cones results in abnormal cystic bulges and waste bags and dispersed active-Caspase-3 signal through the elongated individualizing cysts in the *ago*^*TRiPDf*^ testes (Fig. 2f,g).

DAPI staining showed disturbed nuclear bundles in elongated cysts of *ago*^*ms*^ mutant. (Supplementary Fig. S1 d,d’,e,e’). It was shown previously, that Archipelago has a nuclear localization during larva development^16^. To test the possibility that nuclear phenotype is the consequence of the abnormal chromatin reorganization in the post-meiotic stages, we investigated the displacement of histones by protamines. We found normal Protamine-GFP accumulation, in the nuclei onward of late canoe stage both in control and *ago*^*TRiPDf*^ flies (Fig. 2h,i). Consistently with DAPI staining, many Protamine-GFP positive nuclei are scattered in the elongated cysts (Fig. 2h,i,j,k,l,l’), the nuclei distal to the nuclear bundle, that are scattered along the cysts tail cannot elongate and become hypercondensed.

### Mitochondrial abnormalities in *ago *mutants

**Figure 3:**
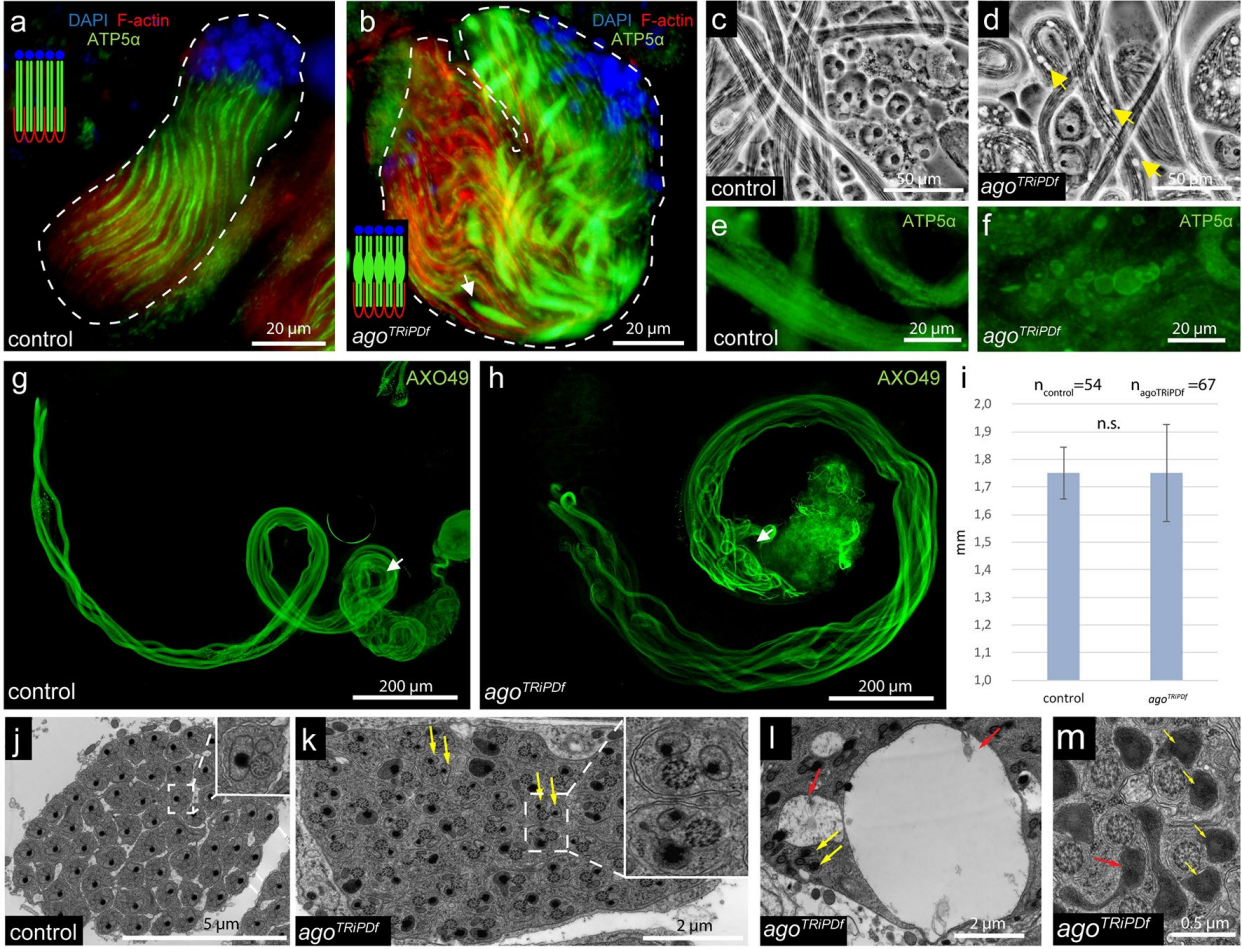
Mitochondrial abnormalities in *ago*^*TRiPDf*^ testis. (**a-b**) ATP5alpha with anti-Atp5 (green), nuclei with DAPI (blue), and investment cones with Phalloidin-TexasRed were visualized on control (**a**) and on *ago*^*TRiPDf*^ (**b**) in early elongating stages. Arrows show mitochondrial bulges next to the elongation complex in *ago*^*TRiPDf*^ cyst(**b**). (**c,d**) Phase-contrast images of elongating cysts in control (**c**) and *ago*^*TRiPDf*^ (**d**) testis with vacuolar structures (arrows). (**e,f**) Anti-ATP5alpha staining on wild type (**e**) and *ago*^*TRiPDf*^ (**f**) of elongated cysts: staining reveals mitochondrial swelling in *ago*^*TRiPDf*^ spermatids. (**g,h**) Anti-AXO49 (green) staining on control (**g**) and on *ago*^*TRiPDf*^ (**h**). Cysts from the *ago*^*TRiPDf*^ testis elongate normally compared to control (**g,h,i**). Statistical significance was determined by Welch two-sample t-test (**i**).( **j-m**) Transmission electron micrographs of wild type (**j**), *ago*^*TRiPDf*^ (**k-m**). We can observe multiple mitochondrial abnormalities in *ago*^*TRiPDf*^, like paracrystalline accumulation in both mitochondrial derivatives (**k,l,m** yellow arrows), mitochondrial swelling (**l**, red arrows) and misshaped mitochondria (**m** red arrow) The charts was created in Microsoft Excel 2016 MSO ver. 16.0.4266.1001 and processed in Adobe Illustrator CS6 ver. 16.0.3.

Several lines of evidence suggest, that mitochondria are the key organelles connected to the spermatid elongation, individualization, and protein degradation in Drosophila^31–33^. Therefore, we tested the mitochondrial structure in *ago*^*TRiPDf*^ flies. First, we visualized the mitochondrial derivatives by anti-ATP5 staining and found that the *ago*^*TRiPDf*^ spermatids showed severe alteration in mitochondrial morphology (Fig. 3a,b). Mitochondrial bulges are present next to the elongation zone at the basal end in *ago*^*TRiPDf*^ elongating cysts. Similar mitochondrial bulges are observable in *parkin*, *clueless* and *fascetto* mutants, which function is described earlier in mitochondria microtubule interactions^31,33^. Furthermore, in elongated *ago*^*TRiPDf*^ cysts we observed large vacuolar structures at the basal end, which we identified as swollen megamitochondria (Fig. 3c–f). This phenotype is mild compared to *bb8*^*ms*^ mutants where the mitochondrial abnormalities result in shorter elongated cysts^29^. To clarify if the observed alterations affect spermatid elongation itself in *ago*^*TRiPDf*^ testis, we measured the length of the late elongated cysts by utilizing AXO49 antibody staining, which identifies the polyglycilated tubulin, representing the fully matured axoneme in the individualizing cysts (Fig. 3g–i)^5^. The mitochondrial alterations do not affect the elongation process of *ago*^*TRiPDf*^ spermatids. To test the ultrastructure of mitochondrial derivatives we utilized transmission electron microscopy on *ago*^*TRiPDf*^ testes. We found normal axoneme formation, however, we observed different mitochondrial abnormalities, which manifest in morphology, size, and paracrystalline material accumulation phenotype (Fig. 3j–m).

These results strongly suggest that Ago activity is necessary for the normal function and organization of post-meiotic mitochondria.

### Ago localization during spermatogenesis

**Figure 4:**
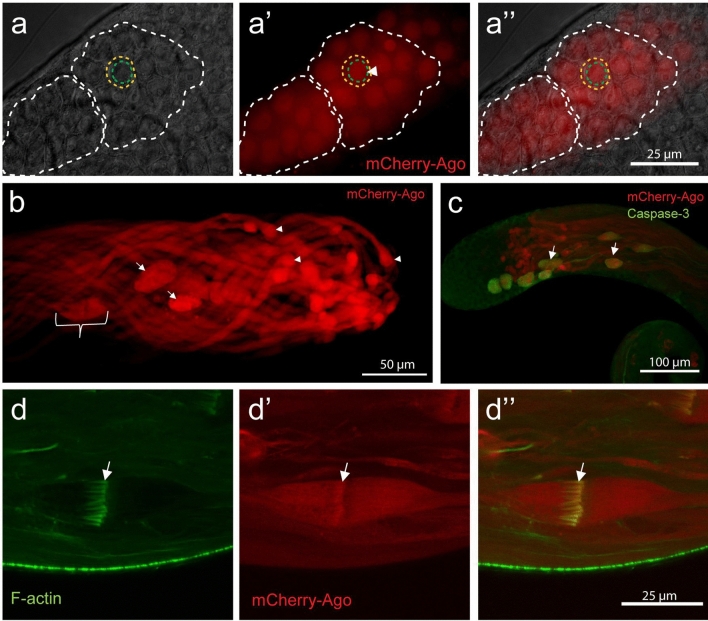
β2-tub-mCherry-Ago localization in testis. (**a,a’,a”**) β2-tub-mCherry-Ago (red) is nuclear in 16-cell cysts (white dashed line; apolar spermatocyte yellow dashed line, nucleus green dashed line). (**b**) After elongation, there is an increasing gradient of mCherry-Ago to the basal end (arrowhead) of the cyst. During individualization, the mCherry-Ago signal localize to the cystic bulges (bracket) and the waste bags (arrows). (**c**) mCherry-Ago signal overlaps with the cleaved Caspase signal (green) in cystic bulges and waste bags. (**d,d’,d’’**) In cystic bulges, the mCherry-Ago signal has a specific enrichment at the base of the actin cones (arrow).

To investigate the potential spatial and temporal presence of Ago function in testis, we established a transgenic fly line with P{β2-tub-mCherry-Ago} (*mCherry-Ago*), where the testis-specific expression of the transgene was driven by the β2-tub promoter^5,34,35^. The functionality of the *mCherry-Ago* was tested in *ago*^*ms*^ background and we found that the transgene was able to partially restore the fertility of *ago*^*ms*^ mutant (Fig. 1d). Next, we investigated the subcellular localization of the transgene and found that mCherry-Ago protein is localized to the nuclei of primary spermatocytes (Fig. 4a,a’,a’’), which is consistent with the localization pattern of Ago in body wall muscle^16^. However, in the elongated spermatids, mCherry-Ago became cytoplasmic with a continuous enrichment towards the basal end of the cysts (Fig. 4b). During individualization, the mCherry-Ago accumulates in the cystic bulges and shows a specific enrichment around the investment cones (Fig. 4b–d,d’,d’’), and after the signal persists in the waste bags (Fig. 4b,c). The stage-specific different localization pattern of Ago suggests a pleiotropic role of it even in spermatogenesis.

## Discussion

Directed protein degradation is a crucial requirement of the spermatid individualization in Drosophila. Multiple data sources suggest, that at least two E3 ligase complexes are involved in caspase activation: the Klhl10/Cul3/Roc1b, and the Ntc/Cul1/SkpA E3 complex^8,13^. Key elements of these complexes are the testis-specific isoform of Cul3 and the testis-specific Ntc proteins. Moreover, it seems the Ntc protein might have a conserved function during spermatogenesis; Fbxo7 the mouse orthologue of Ntc is also required for mouse spermatid development^36^. A previous analysis of the Drosophila testis transcriptome highlighted the existence of several other testis-specific members of the ubiquitin–proteasome system with higher transcript accumulation in later developmental stages, including multiple F-box proteins, and modulator subunits, like the SKP family^9^. All these data suggest the importance of targeted protein degradation in the late stages of spermatogenesis.

Here, we present a previously unknown function of the F-Box protein Ago in the post-meiotic stages of *Drosophila melanogaster* spermiogenesis. Multiple aberrations are present during spermatid differentiation both in classical *ago*^*ms*^ and in the *ago*^*TRiPDf*^ testis. In *ago*^*ms*^ mutant testes, all three *ago* transcript levels are decreased, however, *ago-RA* shows only a moderate decrease, suggesting that *ago-RC* and *ago-RB* could have critical role during spermatogenesis, similarly to what was shown in the case of *ago-RC* function in tracheal differentiation^16^.

We did not observe abnormalities in the early stages of spermiogenesis, however, mCherry-Ago first localizes to the nuclei in primary spermatocytes. The nuclear localization of Ago was demonstrated in ventrolateral body wall muscle, therefore the observed localization in testis suggests a probable redundant function of Ago in the early stages of spermiogenesis^16^. During elongation, the mCherry-Ago protein becomes cytosolic, accumulates at the basal end of the cysts during individualization, and is followed by enrichment in the cystic bulge. Similar to the other known E3 ligase complexes, such as Culin3 and ubiquitin itself, mCherry-Ago also has been transferred to the waste bags^13^. mCherry-Ago localization pattern is also similar to another testis-specific SCF complex member, the Ntc/Cul1/SkpA (SCF^Ntc^)^8^, however in contrast to the SCF^Ntc^, mCherry-Ago is not present in the proximal region of the nuclei, it is localized to the more matured cystic bulges. The co-localization of cleaved caspase signal and mCherry-Ago signal suggests that mCherry-Ago protein might not be a target of caspase activity. *Ago* mutant phenotypes also indicate that Ago is not required for caspase activation. However, it is also tempting to hypothesize that the Ago protein plays role in the restriction of activated caspases to the cystic bulge. This is potentially reinforced by Ago function by restricting the apoptotic activity of the rbf1/de2f1 pathway^15^.

Our observation on mitochondrial abnormalities suggests that Ago protein could play a direct or indirect role in the unfurling and elongation of mitochondrial derivatives. Drosophila Parkin is a member of a ubiquitin ligase complex that was already shown to have a very similar phenotype to *ago*^*TRiPDf*^ testis, with enrichment of mitochondrial bulges in the elongating cysts^7,33^. Direct approaches show the hSel-10 (hAgo, FBW7) protein interacts with Parkin in human neurons and targets CycE^37^. A more recent publication showed that Parkin targets the SCF substrate adapter Fbw7β (hAgo beta isoform) for proteasomal degradation in dopaminerg neurons, where lack of Parkin causes mitochondrial oxidative stress due to the unregulated SCF^Fbw7β^-mediated ubiquitylation-dependent proteolysis of Mcl-1^38^. While Ago was shown to have a role in the oxidative stress response in Drosophila tracheal development^16^, there is no proven interaction between Ago and Parkin in Drosophila. However, the involvement of both Ago and hAgo in oxidative responses raises the possibility of a conserved mechanism, moreover, the similar phenotype of mitochondrial derivatives in *parkin* mutant and *ago*^*TRiPDf*^ testis suggest a potential connection between them in Drosophila as well.

Ago protein role was proposed in growth regulation as an indirect effect on S6kinase (S6K) levels, which place Ago between metabolic signaling and protein synthesis regulation^39,40^. Ago’s role as a metabolic regulator is reinforced by the mitochondrial abnormalities we observed. Furthermore, it was reported the CycE levels depend on mitochondrial morphology^41^, which also makes the Ago a potential link to metabolism since its role in CycE level regulation^24^. The mitochondrial connection is also explicable due to Ago function in hypoxic-induced gene expression^16^. All of these data suggest Ago protein’s role in the differentiation of mitochondrial derivatives during spermatogenesis.

Despite of the abnormal mitochondrial development in *ago*^*TRiPDf*^ testes, there was no measurable elongation defect of the cysts. On the other hand, the integrity of the elongated nuclear bundles was compromised and malformed nuclei were present in the mutants, without effecting the histone-protamine transition. The hyper-condensed nuclei is not exclusive for *ago* mutants, they are characteristic to several male-sterile mutants, such as the *dBruce*, *Mst77F* and *Rae1* mutants^42,43^.

The mitochondrial abnormalities could be sufficient to cause the observed defective individualization complex, however, the mCherry-Ago signal localization in the cystic bulges and in the basal end of elongating cysts suggests a more direct role of Ago in cytoskeletal reorganization, therefore Ago’s function as a mediator between the mitochondria and cytoskeletal elements. In this report, we proposed an additional role of the F-box Ago protein in spermatid differentiation and maturation.

## Materials and methods

### Fly strains and mutants

Fly strains were maintained on standard cornmeal agar medium at 25 °C, or 18 °C. RNAi crosses were incubated at 29 °C up to eclosion. Oregon-R and + */* + *; Df(3L)BSC370/ P{TRiP.HMS00111}attP2ago* was used as wild type control to classical alleles and RNAi knockdowns respectively. The ago^ms^ mutant was obtained from Kyoto DGRC (w^1118^; P{RS5}ago^5-HA-2760^). The ago^1^, ago^3^ mutants were kindly provided by Kenneth H. Moberg (Moberg et al. 2001.) *y*^*1*^, sc^*^ v^*1*^*; P{TRiP.HMS00111}attP2ago,* w^1118^; *Df(3L)BSC370/TM6C, Sb*^*1*^* cu*^*1*^ and w[*]; P{w[+ mC] = protamineB-eGFP}2/CyO fly strains were obtained from Bloomington Drosophila stock center. *bam*-Gal4 testis-specific driver was kindly provided by Helen White-Cooper ^44^. For fertility tests, individual males were crossed with 4–5 virgin Oregon-R females. Males failed to produce offspring after 5 days considered as sterile. Males that produced offspring, but considerably less (~ 50%) than wild type flies, considered semi-sterile.

### Staining and microscopy

Testis preparations and staining were performed as earlier described by White-Cooper, 2004^45^.

4′,6-diamidino-2-phenylindole (DAPI) were used at 1 µg/ml concentration. Texas Red-X Phalloidin and Alexa Fluor 488 Phalloidin (Life Technologies) were used at a 1:250 dilution. Rabbit mAB anti Cleaved Caspase-3 (5A1E Cell Signaling Technology) was used at a 1:200 dilution. Mouse monoclonal AXO49 (Merck) antibody was used at a 1:5000. Mouse anti-ATP5A antibody [15H4C4] (Abcam) was used at a 1:100 dilution. Secondary antibodies, Alexa Fluor 488 and Alexa Fluor 546 (Invitrogen) were used at a 1:400 dilution. SlowFade Gold antifade reagent (Life Technologies) was used as a mountant. Images were taken by using Olympus BX51 fluorescent microscope (Olympus cell^A ver. 3.3 software) or Olympus Fluoview Fv10i Confocal microscope(Olympus FW10-ASW ver. 04.02). The cyst length measurement was conducted as described in Vedelek et al. 2016^29^. Statistical analyses were conducted in R version 3.5.1.

Electron microscopic analysis of testes was done as described in Laurinyecz et al. 2016^46^.

Images were processed with the GIMP 2.8.6.

### qPCR

Total RNA purification was performed from 30 pairs of testes from each genotype, with RNeasy Plus Micro Kit (Qiagen). We utilised the protocol described in Vedelek et al. 2016 for the apical and basal part specific RT-qPCRs^29^. For first-strand cDNA synthesis, RevertAid First Strand cDNA Synthesis Kit (Life Technologies) was used according to the manufacturer’s instruction.

Maxima SYBR Green/ROX qPCR Master Mix (Life Technologies) was used according to manufacturer’s instructions, CFX96 Real-Time PCR Detection System (Bio-Rad) was used with the following reaction conditions: 95 °C 10 min, 50 cycles of 95 °C 15 s, 54 °C 30 s, 72 °C 30 s. *rp49* mRNA was used as a reference. The final values represent the mean and standard error of triplicates. qPCR experiments were conducted on 3 independent biological samples. qPCR primer sequences are available in Supplementary Table S1.

### Molecular biology

β2-tub-mCherry-Ago transgenic construct was established by amplifying *ago* cDNA (BDGP DGC clone LD21322) and mCherry cDNA (mCherry LIC cloning vector, Addgene) with Phusion High-Fidelity DNA Polymerase (New England BioLabs). PCR products were cloned into testis-vector3 (kindly provided by J. A. Brill)^34^. PCR-primer sequences are available in Supplementary Table S1.

## Supplementary Information

Supplementary Information
