# Supplementary material for: The tumor suppressor archipelago E3 ligase is required for spermatid differentiation in Drosophila testis
Source: Sci Rep. 2021 Apr 19;11:8422. doi: 10.1038/s41598-021-87656-3 (PMC8055871; doi:10.1038/s41598-021-87656-3)
Supplement: Supplementary file 1 — Supplementary Information [file 41598_2021_87656_MOESM1_ESM.docx]

**The tumor suppressor archipelago E3 ligase is required for spermatid differentiation in Drosophila testis**

**Viktor Vedelek^1*^, Attila L. Kovács^2^, Gábor Juhász^2^, Elham Alzyoud^1^, Rita Sinka^1*^**

^1^ University of Szeged, Department of Genetics, Szeged, Hungary

^2^ Eötvös Lóránd University of Science, Department of Anatomy, Cell and Developmental Biology, Budapest, Hungary

* Corresponding Authors: Rita Sinka, rsinka@bio.u-szeged.hu, Viktor Vedelek ugu@veta.hu


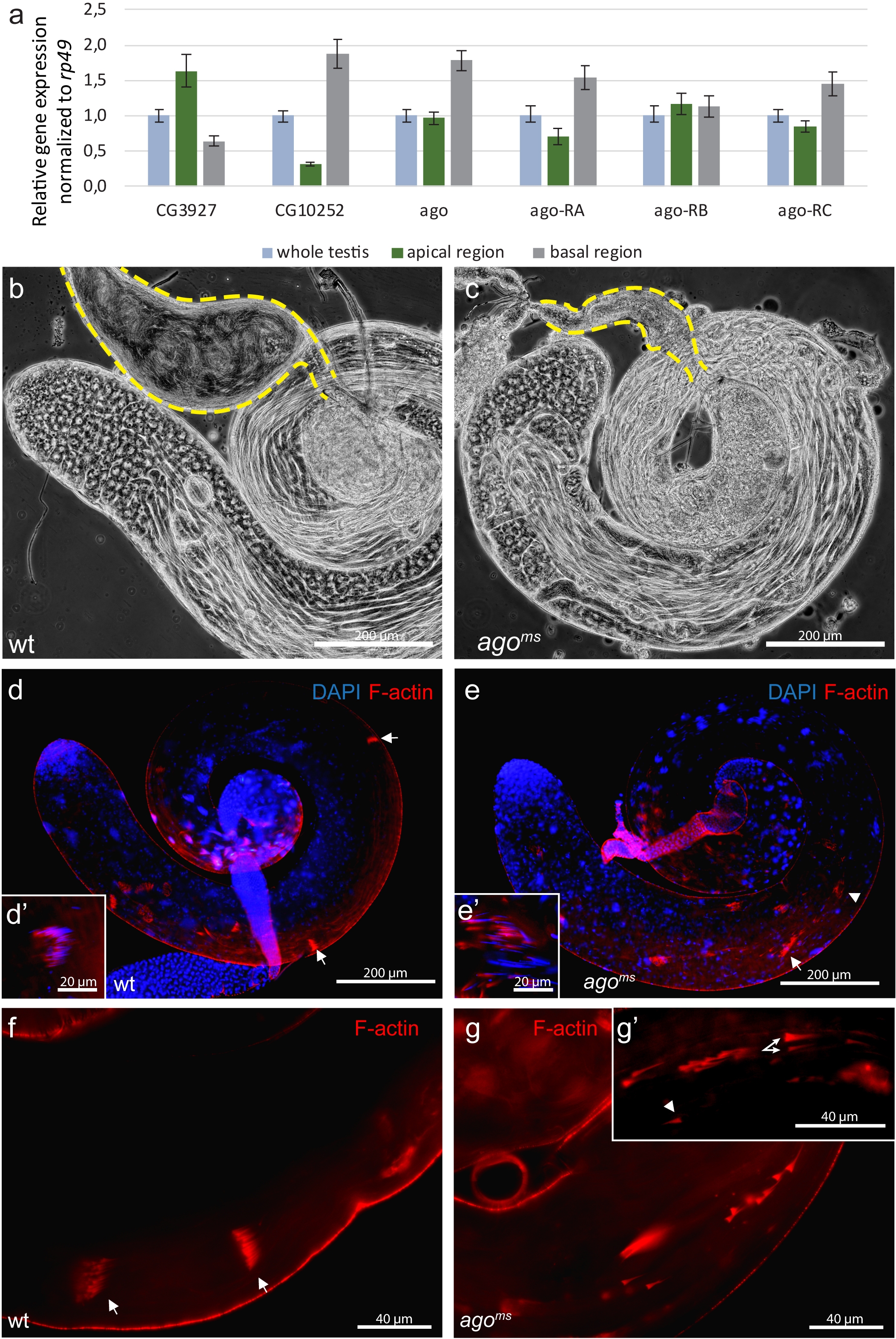


**Fig. S1 *ago* transcript distribution in testis and individualization defects in *ago^ms^* mutants**

(**a**) Relative mRNA expression of *CG3927*, *CG10252*, *ago*, *ago-RA*, *ago-RB* and *ago-RC* in wild type whole testis, apical part and basal part normalized to *rp49*. Measurements were made in triplicates. (**b**, **c**) Phase-contrast image of wild type (WT) and *ago^ms^* mutant testes. Seminal vesicles are highlighted with yellow dashed lines. (**d**-**e**) DAPI staining (blue, **d**, **e**) shows disordered nuclear bundles in *ago^ms^* mutant (**d**’, **e’**). Investment cones were visualized with Texas-Red-Phalloidin (red) in wild type (**d**, arrows, **f**) and *ago^ms^* mutant (**e**, arrows, **g**). Individual actin cones are dispersed in the mutant cysts (**e**, arrows, **g, g’**), and the orientation of them is occasionally damaged (**g’** arrowhead). The chart was created in Microsoft Excel 2016 MSO ver. 16.0.4266.1001 and processed in Adobe Illustrator CS6 ver. 16.0.3.

**Table S1** Primers utilised in the study
